# The characterisation of piRNA-related 19mers in the mouse

**DOI:** 10.1186/1471-2164-12-315

**Published:** 2011-06-15

**Authors:** Harald M Oey, Neil A Youngson, Emma Whitelaw

**Affiliations:** 1 Queensland Institute of Medical Research, Epigenetics, 300 Herston Road, Herston, Brisbane 4006, Australia

## Abstract

**Background:**

Piwi interacting RNA, or piRNA, is a class of small RNA almost exclusively expressed in the germline where they serve essential roles in retrotransposon silencing. There are two types, primary and secondary piRNA, and the latter is a product of enzymatic cleavage of retrotransposons' transcripts directed by the former. Recently, a new class of 19nt long RNA was discovered that is specific to testis and appears to be linked to secondary piRNA biogenesis.

**Results:**

We locate clusters of the testis-specific 19mers, which we call piRNA-related 19mers (pr19RNA), and characterise the transcripts from which they are derived. Most pr19RNA clusters were associated with retrotransposons and unannotated antisense transcripts overlapping piRNA clusters. At these loci the abundance of 19mers was found to be greater than that of secondary piRNAs.

**Conclusion:**

We find that pr19RNAs are distinguished from other RNA populations by their length and flanking sequence, allowing their identification without requiring overlapping piRNAs. Using such sequence features allows identification of the source transcripts, and we suggest that these likely represent the substrates of primary piRNA-guided RNA cleavage events. While pr19RNAs appear not to bind directly to Miwi or Mili, their abundance relative to secondary piRNAs, in combination with their precise length, suggests they may be more than by-products of secondary piRNA biogenesis.

## Background

Piwi-interacting small RNAs (piRNAs) are almost exclusively expressed in the germline where they serve essential roles in retrotransposon silencing [1-5]. There are two types, the primary piRNAs (p-piRNAs) and secondary piRNAs (s-piRNAs), and they associate with three developmentally regulated Ago-related proteins, Miwi, Mili and Miwi2, in mice [6]. The p-piRNAs are identified by their length, spanning ~24-30 ribonucleotides (nt) and the presence of a 5' U. They are mostly derived from long non-coding RNAs transcribed from distinct genomic loci known as piRNA clusters [5,7].

Some p-piRNAs target the transcripts of active retrotransposons and retroviruses, such as the long interspersed nuclear elements (LINEs) and the intracisternal A particles (IAPs). The retrotransposon transcripts are then cleaved enzymatically resulting in the release of s-piRNAs. The initial cleavage site is located within the base-paired region 10 nt downstream of the 5' terminal U of the p-piRNA and the resulting s-piRNAs are therefore distinguished by an A at position 10 [8-10]. Some s-piRNAs are expected to be reverse complementary to the original p-piRNA precursor transcript and may themselves be able to direct cleavage of these to recreate the original p-piRNA. The formation of s-piRNAs directed by p-piRNAs and p-piRNAs directed by s-piRNAs has been been called the ping pong amplification cycle [9]. The ping-pong amplification, identified by the presence of secondary piRNAs, is particularly active at the pre-pachytene stage. At the pachytene stage the population of piRNAs shifts towards p-piRNAs [10,11]. The precise nature of these events in mice is still being investigated and appears to vary according to the complement of Piwi-related proteins that are expressed [10].

In this report we study a class of 19 nt long piRNA-related RNAs (pr19RNA) in mice testes that appears to result from piRNA directed cleavage of transcripts. While this manuscript was in preparation these RNAs were also reported by Berninger *et al*. [12]. However, our analyses differ in that we investigate the genomic origin of most of these 19mers, including those derived from the repetitive IAP and LINE elements, rather than limiting the analyses to non-repetitive RNA. We find that the 19mers are readily distinguished from other RNAs by the composition of their downstream flanking sequence and use this feature to identify likely substrates of piRNA-directed RNA cleavage.

## Methods

### Deep sequencing datasets and initial data processing

Three publicly available spermatogenic tubule small RNA deep sequencing datasets published by Robine *et al *[7]. were obtained from the Gene Expression Omnibus (GEO) [13] under the accession number GSM475279, GSM475280 and GSM475281, and used for the primary analyses. Additionally, small RNA sequencing data from several tissues published by Chiang *et al *[14]. were also obtained under the GEO Series accession GSE20384 and used for some analyses, as specified in the results. Prior to mapping, all reads containing homopolymers > 8 nt were removed and reads matching the partial sequence CCGGGTTTCGGCACC, identified as tRNA-derived, were also removed. In the case of reads mapping to LINE elements, reads matching the patterns (GGA)_4_, (CCT)_4_, (GAA)_4 _and (CTT)_4 _were also removed as these were found to cause mapping artefacts against common simple repeats inside many LINE elements. All other data processing and sequence analyses were carried out using scripts available as part of the Biopieces (http://www.biopieces.org).

### Mapping of reads to LINE and IAP

Reads likely to originate from IAP or LINE elements were identified by mapping the reads to a database containing all mouse IAP elements and one containing all LINE elements annotated in the UCSC Genome Browser RepeatMasker track (accessed 01.09.2010) [15]. To obtain reads likely to originate from IAP or LINE repeats the program Bowtie [16] was used to map reads directly to the IAP and LINE sequences using the options "-v 0 -m 50000" to get high-confidence IAP and LINE small RNA datasets. These reads were then remapped to IAP or LINE consensus sequences with less stringent parameters allowing for two mismatches (Bowtie option -v 2). The consensus sequences that were used were obtained from RepBase [17] under the identifiers "IAPLTR1_MM_LTR" and "IAPLTR1_MM" for IAP and "L1_MM" for LINE. The two IAP reference sequences were joined such that the IAPLTR1_MM sequence was flanked on both sides by a copy of the IAPLTR1_Mm_LTR sequence to obtain an IAP reference sequence that includes LTRs. The sequence composition of pr19RNA and piRNA reads were visualised in the form of sequence logos that were plotted using the "plot_seqlogo" script, which is available as part of the Biopieces (http://www.biopieces.org). The script calculates logos using Shannons general formula for uncertainty [18,19].

### Genome-wide annotation of 19mers

All 19mers from GSM475281 were mapped to the mouse genome (NCBI37/mm9) using Bowtie [16] allowing reads to map to < 6 loci. Reads mapping to multiple loci were randomly assigned to single loci. The sequence of all the reads plus 10nt of downstream flanking sequence was obtained, and the reads were binned according to the identity of the 10^th ^downstream base. The origin of the reads were then found by intersection against UCSC Genome Browser RepeatMasker annotation tracks followed by intersection with piRNA clusters, using 94 piRNA clusters annotated by Lau *et al*. [20], and finally with RefSeq genes, which were also obtained through the UCSC Genome Browser [15].

### Positional relationship of IAP-derived p-piRNAs and s-piRNAs

The tendency of p-piRNAs and s-piRNAs to be positionally fixed to pr19RNAs was investigated by first identifying high-confidence pr19RNAs as well as p-piRNAs and s-piRNAs. The pr19RNAs were defined as reads in same orientation as the IAP reference sequence with the sequence motif N_19_VN_8_A, where N_19 _represents the pr19RNA and V represents any nucleotide except U. The p-piRNAs were reads in the opposite sense of the IAP reference sequence, 24-30 nt in length and with the sequence motif UN_8_BN_n_, where B represents any nucleotide except A. The s-piRNAs were any read in the same orientation as the IAP reference sequence, 24-30 nt in length and with the sequence motif VN_8_AN_n_, where V represents any nucleotide except U. The 5' ends of the s-piRNA 5' ends and p-piRNA 5' ends were then tallied at incrementally increasing distances from pr19RNAs. The positional relationship between these were elucidated by plotting the density of piRNA 5' ends relative to pr19RNA 5' ends, similar to the method employed by Aravin *et al*. [11].

### Distribution of prRNA relative to s-piRNAs across the genome

The pr19RNA, s-piRNAs and p-piRNA sequence patterns were also used to define regions in the mouse genome enriched for pr19RNA and s-piRNAs by selecting those regions where uniquely mapping pr19RNAs outnumber other 19mers, and where uniquely mapping s-piRNAs outnumber p-piRNAs. Regions enriched for both were used to estimate the typical ratio of pr19RNAs relative to s-piRNAs genome-wide.

### Miwi and Mili IP analyses

The IP datasets were mapped to the LINE and IAP consensus sequences as well as miRNAs (miRBase v.16) [21], tRNAs [22], snoRNAs [23,24] and the mitochondrial genome (NCBI37/mm9). The number of mapped reads were then normalised to the number of genomically mappable reads in each library as reads per million.

### Strand-specific real-time quantitative reverse transcription PCR

RNA was extracted from 4-month old C57BL/6 mouse testes with Trizol (Invitrogen), 2 μg was treated with DNase I (New England Biolabs) and used for reverse transcription (RT). RT was carried out using Superscript III (Invitrogen) according to manufacturer's instructions, apart from the following changes; MgCl_2 _was added to a final concentration of 5 mM and the RT step was performed at 50°C. Two strand-specific RT reactions were carried out against each strand of the piRNA cluster using the forward and reverse primers to prime the first-strand synthesis (Target1-forward: GCCTATTGCTCCTTGGACTG, Target1-reverse: AGCTAGCTGAAATCGGATGG, Target2-forward: AGGAAAAAGGTTGCCGATCT, Target2-reverse: CTCTTGAAAGGGCTTCTTGC. GAPDH was reverse transcribed in parallel and used to normalise between the reactions (GAPDH-forward: TCCAGAGACAGCCGCATCT, GAPDH-reverse: ACACCGACCTTCACCATTTTG). Real-time quantitative RT PCR (qRT-PCR) was performed in triplicate using Platinum SYBR Green qPCR mix (Invitrogen) on a Corbett Rotorgene qPCR machine at the default settings. Differences in expression between strands were estimated using the 2^-ΔΔCT ^method [25].

## Results

### Small RNAs derived from IAPs in testis

**Figure 1:**
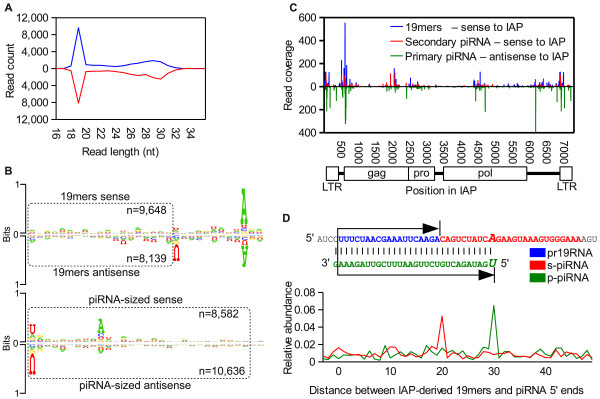
**Features of small RNAs mapped to an IAP consensus sequence (A) The lengths of small RNAs that mapped to IAP are presented**. Reads mapped to the sense strand are presented above the x-axis, and reads mapped in antisense below. (B) The sequence composition of the IAP-derived 19mers and piRNAs were obtained from sequence logos. The reads were separated by their orientation relative to an IAP reference sequence and plotted separately, as indicated. The sequence logos were also extended beyond the 3' ends of the reads (unboxed regions) to reveal any downstream sequence bias. (C) The density of 19mer RNAs mapped to IAP in sense (only reads with an A 10nt downstream and absence of U immediately downstream of the 3' terminus were used), secondary piRNAs mapped to IAP in sense (24-30nt RNAs with A in position 10 and absence of 5' U) and primary-piRNAs mapped to IAP in antisense (24-30nt RNA with 5' terminal U and absence of A in position 10), are plotted. Only the 5' ends of the reads were used to calculate the density. (D) The abundances of IAP-derived p-piRNAs and s-piRNAs, at varying distances from IAP-derived piRNA-related 19mers (pr19RNAs) 5' ends, were tallied and are presented as proportions of the total RNA count.

It is known that p-piRNAs target retrotransposons such as IAPs and LINEs and facilitate cleavage and repression of these genomic parasites in the testes [9,11]. In order to investigate small RNAs with homology to such elements we first mapped small RNAs from a publicly available spermatogenic tubule dataset [7] to the full genomic complement of mouse IAP repeats using stringent alignment parameters. This provided us with a database of IAP-derived reads that were then remapped with less stringent parameters to an IAP consensus sequence. As expected, many of the IAP-derived reads were ~24-30 nt long with sequence features suggesting they were piRNAs (Figure 1A and 1B). However, we also noticed a similarly abundant group of precisely 19 nt long RNAs (Figure 1A). Both the 19mers and the piRNAs were found to map in similar numbers to the sense and the antisense strands of the IAP repeats, a distribution that has been shown previously for the piRNAs and that may be explained by widespread sense and antisense transcription of these elements [11,26]. The 19mers mapped throughout the full length of the IAP repeat, including the long terminal repeat (LTR) regions (Figure 1C). We aligned the 19mers in an effort to identify overrepresented internal nucleotides since many functional small RNAs are found to have such internal preferences [27,28]. No internal patterns emerged and we extended the alignments both upstream and downstream using the IAP reference sequence as a proxy for the original IAP transcripts. These alignments revealed a strong preference for A 10 nt downstream of most 19mers (Figure 1B). Out of the 9,648 19mers that were mapped to the sense strand of the IAP repeat, which has the strongest downstream sequence bias, 7,991 (82%) have an A 10nt downstream, significantly more than the ~25% one would expect by chance. These RNAs are reminiscent of a 19nt long small RNA class recently characterised by Berninger *et al*. [12] using non-repetitive small RNA deep sequencing reads.

Because s-piRNAs have a known preference for A at position 10, it is likely that the 5' ends of the 19mers were derived from a cleavage event 19 nt upstream of the 5' termini of these s-piRNAs. This suggested that many of the 19mers would be flanked by s-piRNAs at their 3' end and overlap with opposite sense p-piRNAs. We tested this hypothesis by tallying the 5' termini of same-sense s-piRNAs and opposite-sense p-piRNAs relative to the 5' ends of the 19mers. As predicted, we found this to be the case (Figure 1D and Additional file 1 Figure S1A). This positional relationship between the 19mers and piRNAs was also described by Berninger *et al*. [12] using non-repetitive reads and it mirrors the relationship between p-piRNAs and s-piRNAs previously described by others [9,11]. The correlation in the distribution of 19mers relative to p-piRNAs and and s-piRNAs is also evident when plotting the density of these along the IAP reference sequence where the 19mers are often found to overlap with same-sense s-piRNAs and opposite sense p-piRNAs (Figure 1C). Out of the 2,743 reads identified as s-piRNAs (RNAs 24-30nt in length with A in position 10 and absence of U in position 1) mapped to the sense-strand of the IAP consensus sequence, 1,931 (70%) were found immediately adjacent to a 19mer (configuration illustrated in Figure 1D). Similarly, out of 4,985 p-piRNAs (RNAs 24-30nt in length with U in position 1 and absence of A in position 10) mapped in antisense to the IAP consensus sequence, 4,434 (89%) were found to have 5' termini located precisely 10 bases downstream of the 3' terminus of a 19mer mapped to IAP in sense (configuration illustrated in Figure 1D).

It did not escape our notice that some 19mers had a strong bias towards U immediately downstream of their 3' ends (Figure 1B). We found that many of these 19mers were positionally locked to the 5' end of same-sense p-piRNAs while they overlapped with opposite sense s-piRNA (Additional file 1 Figure S1A). This suggests that this latter group is formed from enzymatic cleavage of IAP guided by s-piRNAs, thus causing the release of a 19mer with a nucleotide bias characterised by a U immediately downstream of the 3' terminus, instead of an A 10nt downstream. We tallied the number of 19mers that were either immediately adjacent to a piRNA-sized read, or antisense to one, in the two possible configurations indicated in Figure 1D and Additional file 1 Figure S1A, but disregarding whether the piRNAs where p-piRNAs or s-piRNAs. We found that out of the 9,648 19mers mapped to the sense strand, 8,861 (91%) were found with at least one piRNA in such a configuration, while out of 8,139 19mers mapped in antisense, 7,238 (89%) were arranged in this manner. The remaining IAP-derived 19mers have minimal downstream sequence bias, and likely represent RNA degradation products. As the 19mers seem likely to be formed via a piRNA-dependent mechanism, we call them piRNA-related 19mers, or pr19RNAs.

We also investigated the full complement of small RNAs that mapped to any mouse genomic IAP element in our database, as many were not mappable to the consensus IAP sequence. This increased the number of IAP-derived reads two-fold but had only a minor effect on the distribution of 19mers relative to piRNAs (Additional file 1 Figure S1B and S1C). The number of s-piRNAs in the database was 7,700 (defined as reads 24-30 nt in length, the absence of 5'U and the presence of A in position 10), while that of pr19RNAs was 8,839 (defined as 19mers with an A 10nt downstream and absence of U immediately downstream of the 3' termini).

### Small RNAs mapped to LINE elements

**Figure 2:**
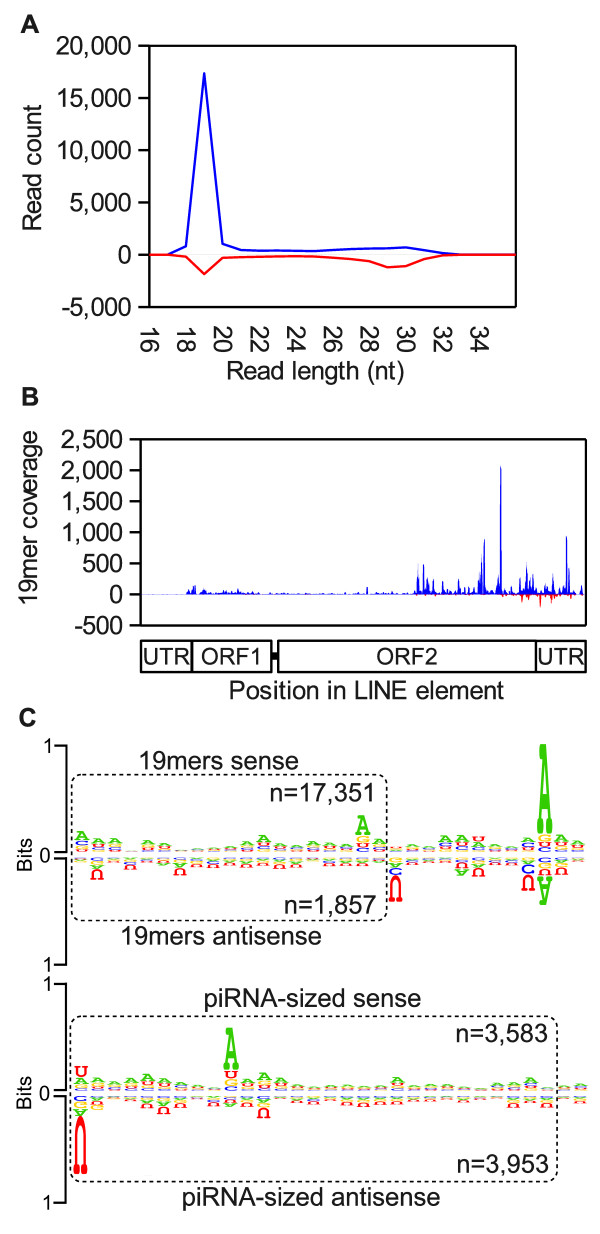
**Distribution of small RNAs mapped to a LINE consensus sequence (A) The lengths of reads mapped to a LINE consensus sequence are presented**. Reads mapped to the sense strand are presented above the x-axis, and reads mapped in antisense below (B) The coverage of 19mers across a 6.4 kb LINE consensus sequence is presented. Reads mapped to the sense strand are plotted above the x-axis, and reads mapped in antisense below. (C) The sequence composition of reads mapped to a LINE consensus sequence are presented. For each type of RNA the reads are separated according to their sense relative to the consensus sequence and plotted separately, as indicated. The sequence logos were extended beyond the 3' ends of the reads (unboxed regions) to reveal any downstream sequence bias.

To ensure that the findings were not specific to IAP, we subjected mouse LINE repeats to similar analyses. We found that the most abundant type of small RNA that mapped to the LINE reference sequence were 19mers with ~17,000 reads mapping almost exclusively to the sense strand of the repeat (Figure 2A and 2B). The combined number of piRNAs was ~7,500, shared equally between the strands. However, we found that the majority of piRNAs that mapped to the sense strand of the repeat had an A in position 10, indicative of s-piRNA biogenesis, while reads mapped to the antisense strand were almost exclusively p-piRNAs, recognised by U in position 1 (Figure 2C). These observations support a mechanism whereby pre-existing p-piRNAs, that are antisense to endogenous LINE transcripts, target these and cause the formation of both s-piRNAs and pr19RNAs. It suggests most LINEs are transcribed in sense and thus only capable of giving rise to sense pr19RNA and sense s-piRNA, unlike IAPs, which have been shown to be transcribed from both strands [11,26]. Like the IAP-derived 19mers, the peak at 19 is clearly defined.

### Most 19mers in mouse spermatogenic tubules are pr19RNAs

**Figure 3:**
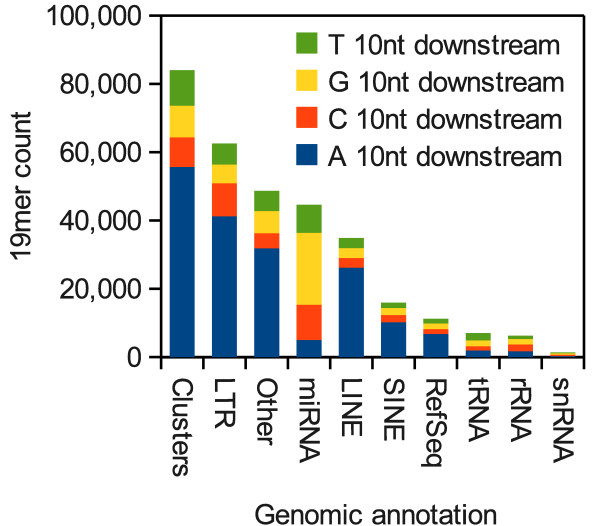
**Genomic annotation of all spermatogenic tubule 19mers The origin of all 19mers from the spermatogenic tubule deep sequencing dataset were deduced from overlap with elements annotated in the UCSC Genome Browser **[15]. The "Clusters" category refers to 94 pachytene piRNA clusters annotated by Lau *et al*. [20] and includes all 19mers mapped to these that were not otherwise annotated. The category "LTR" includes all Long Terminal Repeat sequences, including the Intracisternal A-Particle. Reads that mapped to multiple loci were randomly assigned to single loci.

In total there were 318,960 19mers in the entire small RNA dataset that mapped to regions that were not highly repetitive (defined as having less than 6 mapped loci in the genome). They comprised 142,825 reads mapping to unique loci and 176,135 reads mapping to multiple loci. We investigated the likely origin of all these 19mers by first assigning multi-mapping reads randomly to single loci and then recording the genomic context of all the reads. Using this approach we find that in addition to being derived from retrotransposons, many 19mers are derived from piRNA clusters and refSeq genes and that the majority of these non-repeat associated 19mers also have A 10nt downstream of their 3' termini (Figure 3). A small proportion of 19mers also overlapped with annotated microRNAs and with elements such as rRNAs and tRNAs. However, none of these had any obvious downstream sequence bias and are therefore likely produced via other mechanisms such as RNA degradation (Figure 3). Notably, the small number of tRNA and rRNA-derived reads, typically considered RNA degradation products, suggest degradation is not a major contributor to the 19mer population in the dataset.

### Origin of non-repeat associated 19mers

**Figure 4:**
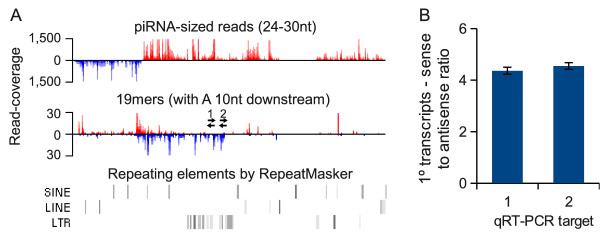
**A piRNA cluster giving rise to both piRNAs and pr19RNAs A) A bidirectional piRNA cluster is presented (NCBI37/mm9 coordinates: chr6:127,734,347-127,787,210)**. The coverage of piRNAs-sized reads (24-30nt) and 19mers (with A 10 nt downstream) is visualised by wiggle-plots with reads mapped from left to right above the x-axis and reads mapped from right to left below. Only uniquely mapping reads are plotted. The position of repetitive elements, adapted from the UCSC Genome Browser [15], is indicated below the plots. The approximate position of two PCR targets used for strand-specific real-time quantitative reverse transcription PCR (qRT-PCR) is also indicated. B) The abundance of the piRNA precursor transcript relative to that of an overlapping antisense precursor transcript is presented. The fold-difference was estimated by strand-specific qRT-PCR targeting two separate loci, as indicated.

As expected from the analyses by Berninger *et al*. [12], the majority of the non-repeat associated 19mers were found to map to p-piRNA clusters. The reads mapped to both strands of these clusters, but with strand-specific differences in their downstream nucleotide composition (Additional file 1 Figure S2). Many of those in the same orientation have a 5' U and minimal downstream sequence bias, suggesting they represent p-piRNA degradation products, while most reads in the opposite sense were found to have a bias towards A 10nt downstream of their 3' termini. By analysing specific piRNA clusters we find evidence of a reciprocal distribution of pr19RNAs and p-piRNAs, suggesting the antisense 19mers originate from unannotated antisense transcripts (Figure 4A). We validated the presence of one such unannotated transcript using qRT-PCR and find that the abundance of the antisense transcript is ~25% that of the sense transcript (Figure 4B).

**Figure 5:**
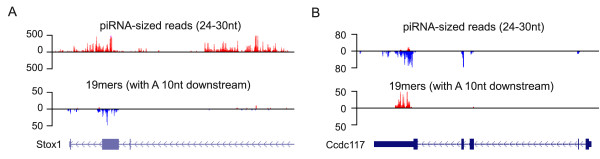
**Genes overlapped by piRNAs and 19mers (A) A piRNA cluster, transcribed from left to right and overlapped by a gene transcribed in the opposite direction, is presented (NCBI37/mm9 locus: chr10:62,120,321-62,152,434)**. The coverage of piRNA-sized reads (24-30nt) and 19mers (with A 10 nt downstream) is also presented in the form of wiggle plots with reads mapped from left to right above the x-axis and reads mapped from right to left below. Only uniquely mapping reads were used. The position of a gene (Stox1) is indicated with exons represented by filled boxes, arrows indicating the direction of transcription and the thin boxes representing the untranslated regions (UTRs). (B) A gene giving rise to piRNAs and and opposite sense 19mers is presented (NCBI37/mm9 locus: chr11:5,428,021-5,442,615). The coverage of piRNAs-sized reads (24-30nt) and 19mers (with A 10 nt downstream) is plotted in the form of wiggle plots with reads mapped from left to right above the x-axis and reads mapped from right to left below. Only uniquely mapping reads were used. The position of the gene (Ccdc117) is indicated with exons represented by filled boxes, arrows indicating the direction of transcription and thin boxes representing the UTRs.

We also used the presence of pr19RNAs to locate genes that appear to be targeted by p-piRNAs. One such gene was found to overlap a p-piRNA cluster in the opposite sense with 19mers confined to an exonic region. Such a distribution suggests the 19mers originate from p-piRNA-directed cleavage of the mRNA (Figure 5A). In many other cases mRNAs were themselves found to give rise to p-piRNAs while the 19mers were found in the opposite orientation suggesting the 19mers may originate from antisense transcripts with cleavage directed by the genic p-piRNAs (Figure 5B and Additional file 1 Figure S3).

**Figure 6:**
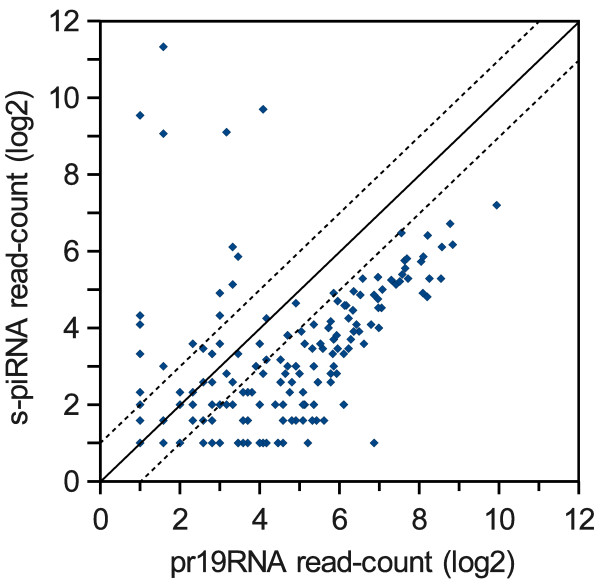
**Abundance of pr19RNA relative to s-piRNAs The number of piRNA-related 19mers (pr19RNA) and secondary piRNAs (s-piRNAs) overlapping 2,5kb non-overlapping tiles across the genome were plotted**. Only tiles overlapping both pr19RNA and s-piRNAs in the same sense were used and tiles with abundant p-piRNAs on the same strand were excluded.

At all the genomic loci that were investigated pr19RNAs were found to be more abundant than s-piRNAs. To test this observation on a larger scale we investigated all regions in the mouse genome enriched for both 19mers and s-piRNAs. These regions were obtained by splitting the genome into 2.5 kb tiles and calculating ratios of different types of uniquely mapping RNAs overlapping each tile. When plotting the abundance of pr19RNAs against the abundance of s-piRNAs we find that pr19RNAs are generally more than twice as abundant as s-piRNAs (Figure 6). Performing a two-sided paired Wilcoxon rank test on these values produces a p-value of 2.2 × 10^-16^. Of the small number of outliers that favour the s-piRNAs, most were caused by abundant reads from point sources within p-piRNA clusters and likely represent atypical p-piRNAs (lacking U in position 1) rather than s-piRNAs. The greater abundance of pr19RNAs across these non-repetitive loci is consistent with the observations at IAP and LINE repeats, where pr19RNAs were also found to be more abundant than s-piRNAs.

### Little evidence for binding of pr19RNA to Miwi or Mili

**Figure 7:**
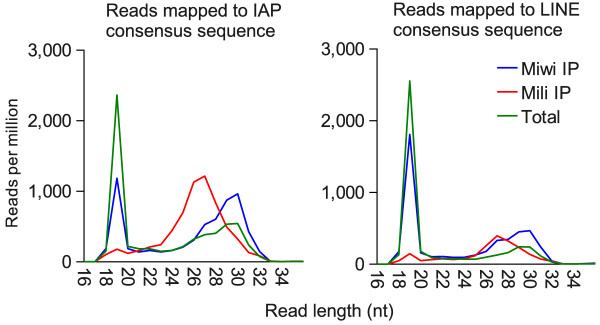
**Miwi and Mili IP small RNA derived from IAP and LINE Deep sequencing reads from co-immunoprecipitated (IP) small RNAs were mapped to an IAP consensus sequence and a LINE consensus sequence**. Each plot was normalised to reads per million mappable reads in the library.

We also investigated the presence of 19mers in the Miwi and Mili co-immunoprecipitated (IP) small RNA datasets from Robine *et al*. [7], focussing on reads mapped to IAP and LINE elements. As expected, we find that both IP libraries are enriched for ~26mers (Mili IP) and ~30mers (Miwi IP) (Figure 7). However, the peaks at 19nt from the IP libraries are lower than that from the total lysate library, suggesting these are not appreciably enriched. The peaks at 19nt observed in the IP libraries may be caused by non-specific contamination, rather than association with Mili or Miwi. To investigate this further we calculated the proportion of reads derived from miRNAs, snoRNAs, mitochondria and tRNAs in the IP libraries and the total lysate library (Additional file 2). RNAs derived from these RNA classes were used as they are not expected to bind Miwi or Mili directly. We find that the amount of RNA from miRNAs, snoRNAs, mitochondria and tRNAs in the IP libraries relative to the total lysate library (Additional file 1 Table S1), is similar to the relative amount of 19mers in these libraries (Figure 7). This is consistent with previously published experimental results where radioactively labelled Miwi and Mili IP RNA run on gels show no evidence of bands at 19nt [11,29].

### pr19RNAs in somatic tissues

**Figure 8:**
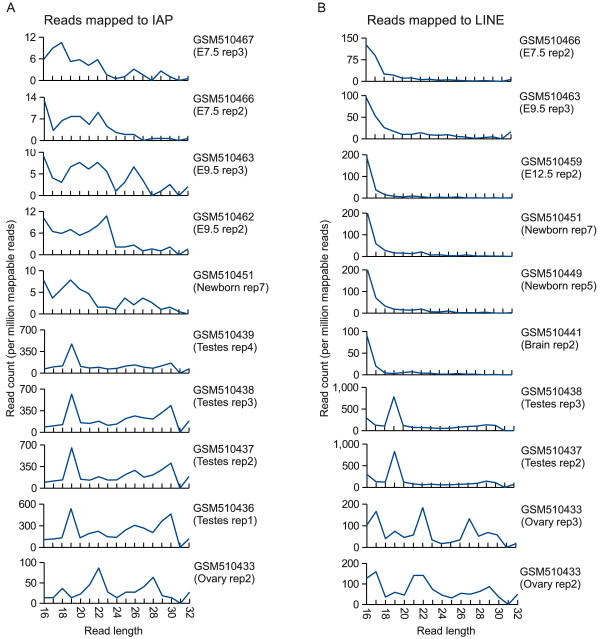
**Distribution of LINE and IAP-derived small RNA across tissues Small RNAs from a publicly available dataset published by Chiang *et al*. **[14]**were mapped to A) an IAP consensus sequence and B) a LINE consensus sequence, and the length of the mapped reads were plotted**. Only Libraries with > 100 mappable reads were included, and in the case of the LINE repeat only a representative selection of replicates from tissues and embryonic time-points are shown. The NCBI GEO [13] accession number and associated tissue annotation is shown for each dataset.

We also extended our analysis to somatic tissues in order to test whether the pr19RNAs are limited to testes. For this purpose we mapped deep sequencing reads from a public small RNA dataset, which included several somatic tissues in addition to testis [14], to the IAP and LINE consensus sequences. While reads from the testis libraries produce distinct peaks at 19nt, reads from the remaining tissues produce no such peaks (Figure 8). The IAP and LINE-derived 19mers therefore seem likely to be testis specific, as concluded by Berninger *et al*. [12] in their analysis of non-repetitive RNA.

## Discussion

In this report we characterise novel members of a recently discovered class of 19nt long RNAs that originate from piRNA-directed RNA cleavage. In a previous paper Berninger *et al*. [12] elegantly show that these small RNAs can be distinguished by their spatial relationship to p-piRNAs and s-piRNAs. As expected, we find that this class of RNA is readily distinguished from other RNAs by the composition of the downstream flanking sequence. By making use of sequence composition rather than overlap with piRNAs a greater number of pr19RNAs can be identified. Their precise length suggests they are formed from two separate enzymatic cleavage events with the 3' end likely to be linked to the formation of s-piRNA 5' ends and the 5' end likely to be representing a novel event. The biogenesis of s-piRNAs has been hypothesised to require only a single enzymatic cleavage event at the 5' end, with the 3' end defined by a less specific mechanism resulting in their variable lengths [9].

Curiously, the IAP and LINE elements, as well as the reverse strand of many piRNA clusters, were found to have a greater number of associated pr19RNAs than s-piRNAs. This difference is puzzling as their biogenesis appears linked and the latter is expected to accumulate more readily due to their association with Miwi and Mili. It may suggest that subsequent steps in the formation of these RNAs are not synchronised. Alternatively, pr19RNAs may themselves be protected from degradation via a mechanism independent of Miwi or Mili as neither appear to associate strongly with pr19RNAs. The abundance of pr19RNAs mappable to the LINE consensus sequence is particularly interesting as they also outnumber p-piRNAs. It has been shown that miRNAs are incorporated into multiple-turnover enzymes such that a single microRNA can guide several consecutive cleavage events [30]. If p-piRNAs function in a similar manner, then the number of pr19RNAs would depend on the abundance of substrate (e.g. LINE transcripts) rather than the abundance of p-piRNAs.

By locating regions enriched for 19mers with an A 10nt downstream we find several genes with a reciprocal pattern in the distribution of 19mers and p-piRNAs. The transcripts from some of these genes appear to be the the target of piRNA-guided cleavage resulting in release of a 19mer. A similar mechanism where p-piRNAs target the transcript of a protein-coding gene was recently proposed for piRNAs in *Drosophila *[31]. This process appears analogous to the endogenous short interfering RNAs that operate in some mammalian tissues [28,32,33]. However, most pr19RNAs co-localising with genes were found to occur in the opposite sense. These 19mers likely arise when genic piRNAs, which are abundant in testis [7], direct the cleavage of overlapping antisense transcripts. The purpose of such events is not immediately clear.

## Conclusion

Based on the sequence composition of the pr19RNA flanking regions and the positional relationship of pr19RNAs to p-piRNAs and s-piRNAs, we conclude that pr19RNAs are produced by enzymatic cleavage events guided mainly by p-piRNAs in adult mouse spermatogenic tubules. This was also the conclusion reached by Berninger *et al*. [12] in their analyses of overlapping piRNAs and pr19RNAs. Like many newly described classes of small RNAs, a function for pr19RNAs has not yet been ascertained. However, the 5' cleavage site, precise length and abundance of these RNAs suggest the underlying mechanism serves an important role in piRNA-directed post-transcriptional gene regulation. The characterisation of this novel class of small RNA offers new insight into the biogenesis and function of piRNAs in the germline and is likely to facilitate new methods for elucidating the targets of these enigmatic small RNAs.

## Authors' contributions

HMO carried out bioinformatics analyses and NAY carried out the experimental work. HMO and EW designed the study. HMO, NAY and EW wrote the manuscript. All authors read and approved the final manuscript.

## Supplementary Material

Additional file 1 **Supplementary Figures**. Click here for file

Additional file 2 **Proportion of non-piRNA reads in IP libraries**. Click here for file
