# Supplementary material for: The characterisation of piRNA-related 19mers in the mouse
Source: BMC Genomics. 2011 Jun 15;12:315. doi: 10.1186/1471-2164-12-315 (PMC3143105; doi:10.1186/1471-2164-12-315)
Supplement: Additional file 1 — Supplementary Figures. [file 1471-2164-12-315-S1.DOC]

**A**

ID Count Alignment

00904620 6 -GCUAAUUUGUCAAAAAGUC--------------------------------------------------

01547214 21 ----------UCAAAAAGUCUUUUUCAGU-----------------------------------------

01547213 12 ----------UCAAAAAGUCUUUCUCAGU-----------------------------------------

00392013 11 -----------------------------AUAUGUUACAGAAUUGGAUGGCUGAAUUU------------

00392010 6 -----------------------------AUAUGUUACAGAAUUGGAUGGCUGAAU--------------

00391996 15 -----------------------------AUAUGUUACAGAAUUGGACGGCUGAAUUU------------

01519315 15 ------------------------------UAUGUUACAGAAUUGGAUGGCUGAAUUU------------

01519314 7 ------------------------------UAUGUUACAGAAUUGGAUGGCUGAAUU-------------

Reads mapped to IAP in sense (reads in 5' to 3' direction)

01519298 11 ------------------------------UAUGUUACAGAAUUGGACGGCUGAAUUUG-----------

00062002 8 -------------AAAAGUCUUUUUCAGUAUA--------------------------------------

02376907 72 --------------------------------UGUUACAGAAUUGGAUGGCUGAAUUUGA----------

02376906 25 --------------------------------UGUUACAGAAUUGGAUGGCUGAAUUUG-----------

02376904 49 --------------------------------UGUUACAGAAUUGGAUGGCUGAAUUU------------

02376880 92 --------------------------------UGUUACAGAAUUGGACGGCUGAAUUUGA----------

02376879 33 --------------------------------UGUUACAGAAUUGGACGGCUGAAUUUG-----------

02376876 35 --------------------------------UGUUACAGAAUUGGACGGCUGAAUUU------------

02431979 6 ----------------------------------UUACAGAAUUGGAUGGCUG-----------------

00269017 6 --------------------------------------AGAAUUGGAUGGCUGAAUUUG-----------

00269003 7 --------------------------------------AGAAUUGGACGGCUGAAUUUGU----------

IAP consensus

00232389 7 ----------------------------------------------ACGGCUGAAUUUGAACAGA-----

**AGCUAAUUUGUCAAAAAGUCUUUUUCAGUAUAUGUUACAGAAUUGGACGGCUGAAUUUGAACAGAUCCUU**

00781592 15 -CGAUUAAACAGUUUUUCAG--------------------------------------------------

00389517 8 --------ACAAUUUUUCAGAAAGAGUCAUAUA-------------------------------------

01142014 10 ---------CAAUUUUUCAGAAAGAGUCAUAUACAAU---------------------------------

00991851 9 ---------CAAUUUUUCAGAAAGAGUCAUAUACAAUG--------------------------------

00176018 5 --------------------------------------UCUUAACCUGCCGACUUAA-------------

00389516 26 ---------CAAUUUUUCAGAAAGAGUCAUAUA-------------------------------------

02274826 42 --------ACAAUUUUUCAGAAAGAGUCAUAUACAAUGU-------------------------------

02274825 243 ---------CAAUUUUUCAGAAAGAGUCAUAUACAAUGU-------------------------------

02274806 18 ---------CAGUUUUUCAGAAAAAGUCAUAUACAAUGU-------------------------------

Reads mapped to IAP in antisense (reads in 3' to 5' direction)

02274822 65 ----------AAUUUUUCAGAAAGAGUCAUAUACAAUGU-------------------------------

02274821 28 -----------AUUUUUCAGAAAGAGUCAUAUACAAUGU-------------------------------

02274820 14 ------------UUUUUCAGAAAGAGUCAUAUACAAUGU-------------------------------

00091356 6 ---------------------------------------CUUAACCUGCCGACUUAAA------------

00686679 7 ---------CAAUUUUUCAGAAAGAGUCAUAUACAAUGUC------------------------------

00686676 16 ------------UUUUUCAGAAAGAGUCAUAUACAAUGUC------------------------------

00686674 12 -------------UUUUCAGAAAGAGUCAUAUACAAUGUC------------------------------

00464985 9 ----------------------------------------UUAACCUGCCGACUUAAAC-----------

00389513 5 ------------UUUUUCAGAAAGAGUCAUAUA-------------------------------------

02549211 7 -----------UUUUUUCAGAAAGAGUCAUAUACAAUGUCUU----------------------------

02549208 29 ------------UUUUUCAGAAAGAGUCAUAUACAAUGUCUU----------------------------

02549205 6 -------------UUUUCAGAAAGAGUCAUAUACAAUGUCUU----------------------------

02472388 13 ------------------------------------------AACCUGCCGACUUAAACUU---------

19mer

Consensus

Sense 5' UCAAAAAGUCUUUUUCAGUAUAUGUUAC**A**GAAUUGGACGGCUGAAUUU 3'

IAP **AGCUAAUUUGUCAAAAAGUCUUUUUCAGUAUAUGUUACAGAAUUGGACGGCUGAAUUUGAACAGAUCCUU**

Antisense 3'CAAUUUUUCAGAAAGAGUCAUAUACAAUG**U**CUUAACCUGCCGACUUAAA 5'

Secondary piRNA

19mer

Primary piRNA

**B**


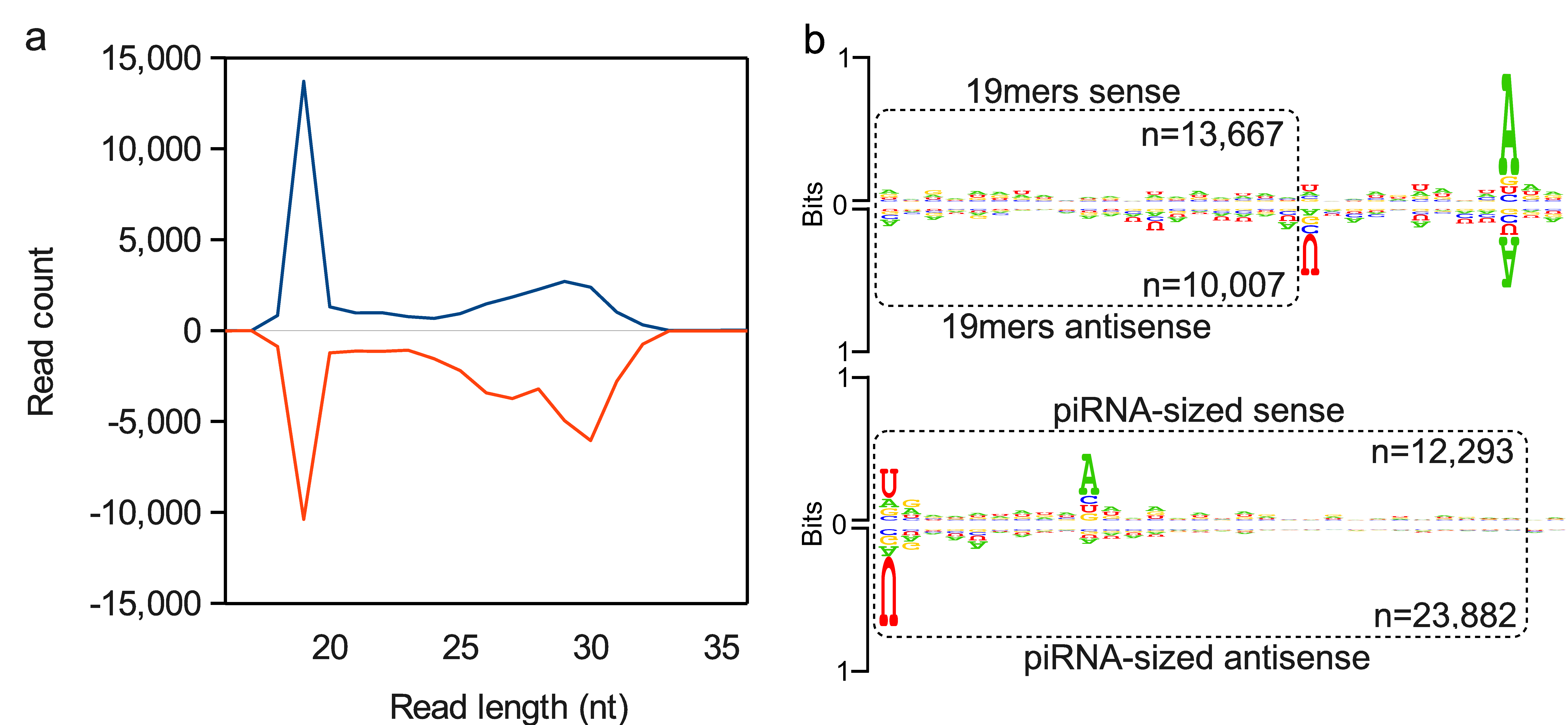
 19mer U position 1 A position 10

**C**

Figure S1. PiRNAs and 19mers mapped to IAP elements

(A) A region of IAP overlapped by primary piRNAs (recognised by 5'U), secondary piRNAs (recognised by A in position 10), 19mers and some unidentified small RNAs, is presented. The number of times each sequence was cloned is presented in column two and notable features are highlighted. Reads with counts lower than five were omitted and some reads were also removed for the sake of clarity. A consensus showing the proposed relationship between the 19mers, and the primary and secondary piRNAs is also presented. (B) Many IAP-derived reads could not be aligned to a single IAP consensus sequence and the full complement of reads mapped to genomic IAPs were therefore also investigated. The length distribution of all these reads, separated according to orientation relative to the IAP element, were plotted showing a distinct peak at 19 nt. The lengths were plotted separately for reads mapped in sense (above x-axis) and reads mapped in antisense (below x-axis). (C) The sequence of the 19mers and the piRNA sized reads are presented in the form of sequence logs. The reads were separated by their orientation relative to an IAP reference sequence and plotted separately, as indicated. The sequence logos were also extended beyond the 3' ends of the reads (unboxed regions) to reveal any downstream sequence bias. The 19mers have a strong preference for A 10 nt downstream of their 3' termini. A subset of the prRNAs, mostly mapped to IAP in antisense, were found to have a distinct preference for U immediately downstream of their 3'end. The piRNAs were found to be a mix of primary piRNAs (recognised by a 5' U) and secondary piRNAs (recognised by A in position 10).


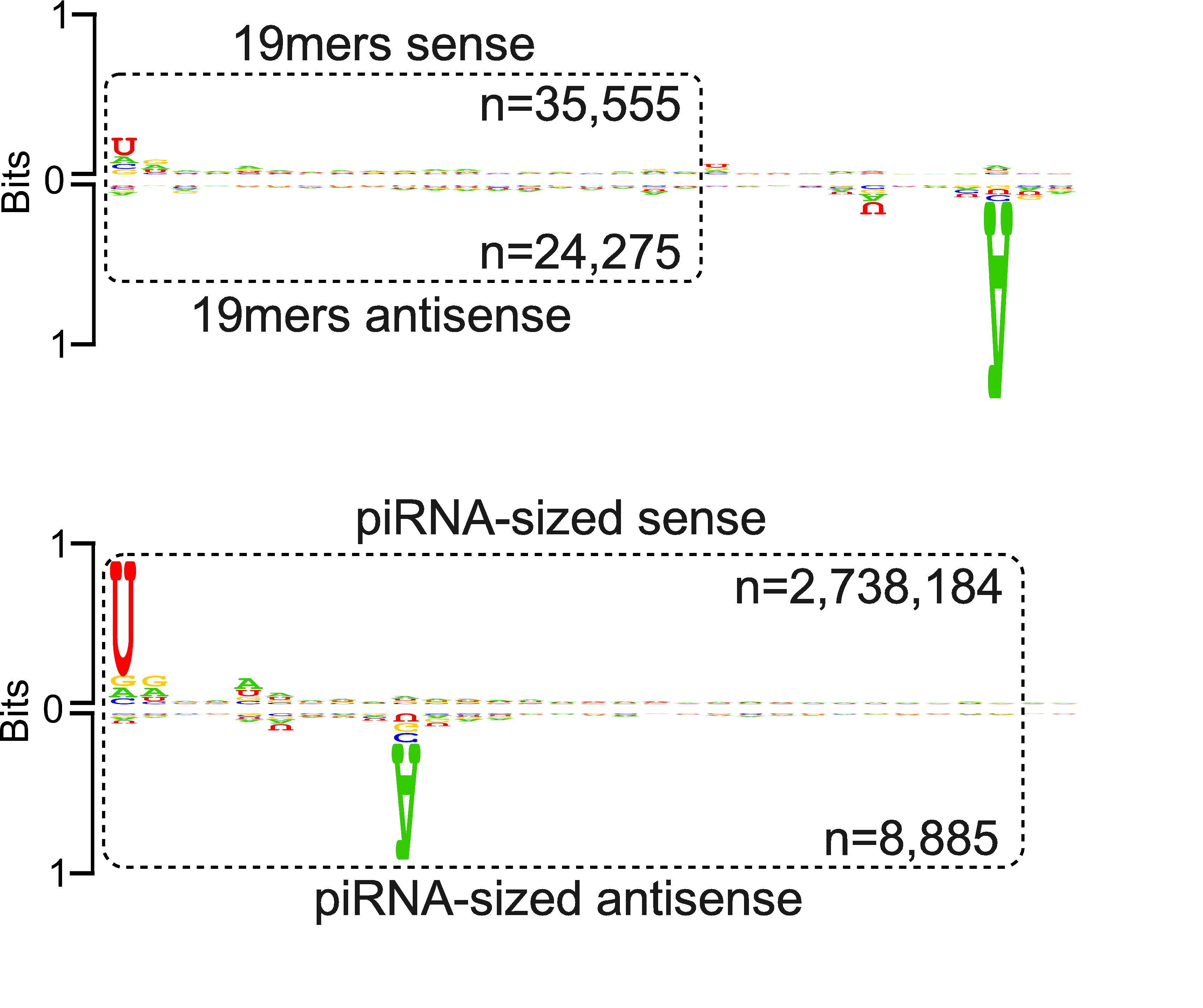


Figure S2. Sequence composition of reads overlapping piRNA clusters.

The sequence composition of piRNA sized reads (24-30nt) and 19mers overlapping the 25 most prolific piRNA clusters annotated by Lau *et al.* [1] are presented. The prevailing direction of piRNAs reads were used to determine the strandedness of each cluster and separate logos were created for reads that mapped in sense or antisense, as indicated. The sequence logos were extended beyond the 3' ends of the reads (unboxed regions) to reveal any downstream sequence bias. Only reads that mapped to single genomic loci were used to generate these plots.


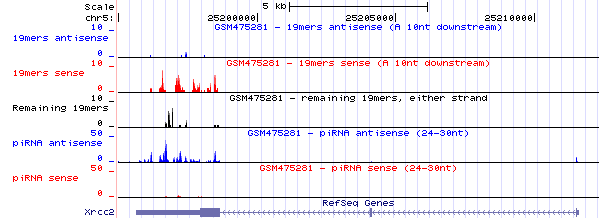


Locus: chr5:25,194,982-25,212,329


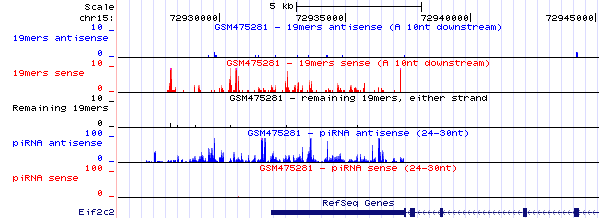


(Locus: chr15:72,925,932-72,945,130)


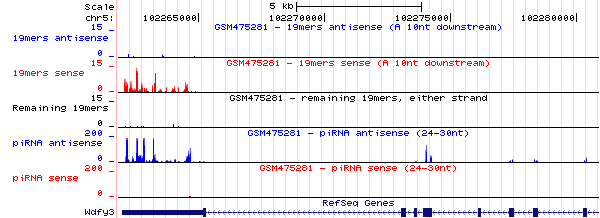


(Locus: chr5:102,261,811-102,280,907)


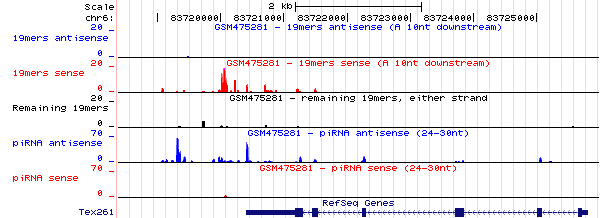


Locus: chr6:83,718,377-83,725,987


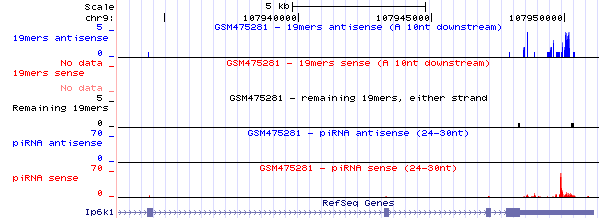


Locus: chr9:107,933,239-107,951,285


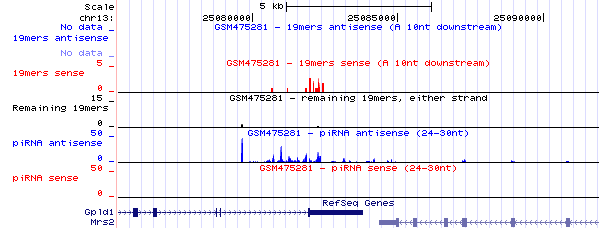


(Locus: chr13:25,075,389-25,091,913)


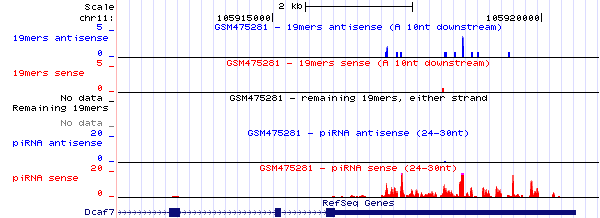


(Locus: chr11:105,912,133-105,921,063)

Figure S3. Genes enriched for primary piRNAs and prRNAs

Several genes that were found to be overlapped by both primary piRNAs and prRNAs are presented by UCSC Genome Browser [2] screen shots. The overlapping small RNAs are plotted separately according to the type of RNA and the direction of the reads with reads mapped from right to left defined as antisense and reads mapped from left to right defined as sense. The RNA coverage is shown in the form of wiggle plots with read-depth indicated on the y-axes. The prRNAs were defined as all 19mers with an A downstream in position +29 and those 19mers that did not fit this pattern are plotted separately, as indicated. The exons of genes in the regions are indicated by filled boxes, the arrows indicate the direction of transcription and the thin boxes at the 3'ends represent the untranslated regions (UTRs).

References

1 Lau, N.C., Seto, A.G., Kim, J., Kuramochi-Miyagawa, S., Nakano, T., Bartel, D.P. and Kingston, R.E.: Characterization of the piRNA complex from rat testes. Science 2006, 313:363-367.

2 Rhead B, Karolchik D, Kuhn RM, Hinrichs AS, Zweig AS, Fujita PA, Diekhans M, Smith KE, Rosenbloom KR, Raney BJ, Pohl A, Pheasant M, Meyer LR, Learned K, Hsu F, Hillman- Jackson J, Harte RA, Giardine B, Dreszer TR, Clawson H, Barber GP, Haussler D, Kent WJ: The UCSC Genome Browser database: update 2010. Nucleic Acids Res 2010, 38:D613-9.
