# Supplementary material for: The characterisation of piRNA-related 19mers in the mouse
Source: BMC Genomics. 2011 Jun 15;12:315. doi: 10.1186/1471-2164-12-315 (PMC3143105; doi:10.1186/1471-2164-12-315)
Supplement: Additional file 2 — Proportion of non-piRNA reads in IP libraries. [file 1471-2164-12-315-S2.DOC]

Proportion of non-piRNA reads in IP libraries

The number of reads derived from RNA-classes expected to have minimal affinity towards Mili or Miwi are presented. The proportion of these reads in the co-immunoprecipitated RNA (IP) libraries relative to the same proportion in the total spermatogenic tubule lysate library is also presented.
